# A case report of cryptococcal meningitis associated with ruxolitinib

**DOI:** 10.1097/MD.0000000000019587

**Published:** 2020-03-27

**Authors:** Daisuke Tsukui, Hiroaki Fujita, Keisuke Suzuki, Koichi Hirata

**Affiliations:** Department of Neurology, Dokkyo Medical University, Mibu, Tochigi, Japan.

**Keywords:** cryptococcus, immunosuppression, Janus kinase inhibitor, meningitis, ruxolitinib

## Abstract

We herein report a 76-year-old Japanese man with myelofibrosis who developed cryptococcal meningitis. After treatment for 5 months with ruxolitinib, the patient presented with fever and disturbance of consciousness. Marked nuchal stiffness was noted. The magnetic resonance imaging results of the brain were normal. Lumbar puncture showed an opening cerebrospinal fluid (CSF) pressure of 110 mm H_2_O, pleocytosis (85 mononuclear cells and 222 polymorphonuclear cells/μL), decreased CSF/serum glucose ratio (43%), and elevated protein (194 mg/dL). Blood and CSF cultures grew no bacteria or fungi. However, cryptococcal antigen was detected in the blood and CSF samples. We discontinued ruxolitinib and started administration of amphotericin B. His condition improved gradually 1 week after initiation of treatment. There have been only a few reports on cryptococcal meningitis associated with ruxolitinib. Physicians should consider the possibility of cryptococcal meningitis in patients receiving ruxolitinib.

## 1 Introduction

Ruxolitinib, an inhibitor of Janus kinase (JAK) 1 and 2, has been approved for the treatment of myelofibrosis (MF) and polycythemia vera (PV) by reducing spleen size, ameliorating debilitating symptoms, and improving overall survival.^[1,2]^ The JAK/signal transducer and activator of transcription (STAT) pathway is the principal signaling mechanism for numerous cytokines and growth factors. JAK inhibitors exert immunosuppressive activities through the downregulation of several cytokines, such as interleukins, interferon-γ, and tumor necrosis factor-α,^[3]^ and result in dysfunction of dendritic cells (DCs),^[4]^ T-regulatory cells,^[5]^ and natural killer (NK) cells.^[6]^ Cryptococcal meningitis is known to occur particularly frequently in immunocompromised hosts.^[7]^ However, there have been only 2 reports of cryptococcal meningitis in patients treated with JAK inhibitors. Here, we report a case of cryptococcal meningitis in a ruxolitinib-treated patient with primary MF.

## 2 Case report

**Figure 1:**
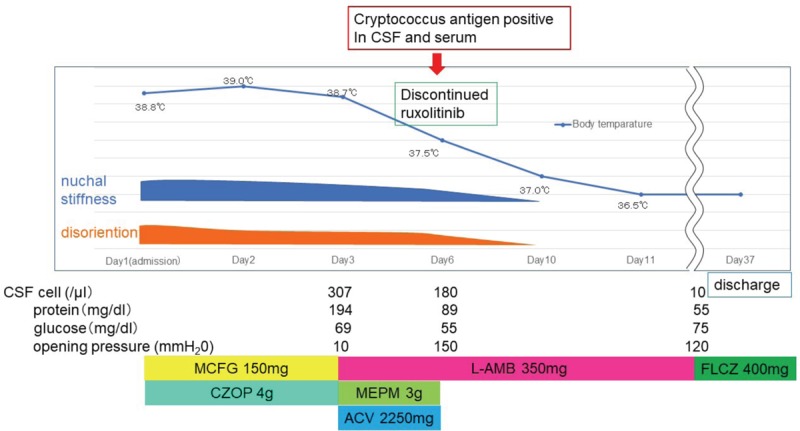
Clinical course of the present case. ACV = acyclovir, CSF = cerebrospinal fluid, CZOP = cefozopran, FLCZ = fluconazole, L-AMB = amphotericin, MCFG = micafungin, MEPM = meropenem.

At the age of 64 years, the present patient was diagnosed with essential thrombocythemia, and hydroxycarbamide was initiated. His condition had been stable for several years. At the age of 72 years, anagrelide hydrochloride hydrate was started instead of hydroxycarbamide because of worsening thrombocytosis. In February 2019, the patient was diagnosed with MF according to the results of bone-marrow puncture. Although the JAK2 mutation was negative, the myeloproliferative leukemia virus oncogene (MPL) W515L mutation was detected. Since then, he had been treated with ruxolitinib (10 mg/d). At the age of 76 years (5 months after initiation of ruxolitinib), the patient was admitted to our hospital because of high-grade fever and disturbance of consciousness from a day before admission. On examination, his body temperature was 38.8°C; his other vital signs were normal. Marked nuchal stiffness was noted. The patient was disoriented to time and place. Cranial nerves were intact. There was no motor weakness or cerebellar ataxia. Tendon reflexes were normal and symmetrical without any pathological reflexes. No sensory impairment was noted. Laboratory data showed mildly elevated C-reactive protein levels (0.31 mg/dL) and procalcitonin levels (0.10 ng/mL). Markedly elevated ferritin levels (2203.5 ng/mL) were observed. The white blood cell count (7000/μL) and platelet count (25.9 × 10^4^/μL) were preserved, but the red blood cell count was decreased (227/μL). Normal levels of β-d-glucan were observed (6.0 pg/mL). Lumbar puncture yielded an opening cerebrospinal fluid (CSF) pressure of 110 mm H_2_O and pleocytosis (85 mononuclear cells and 222 polymorphonuclear cells/μL). The CSF glucose level was 69 mg/dL with a low CSF/serum glucose ratio of 43%, and the protein level (194 mg/dL) was elevated. Herpes simplex virus DNA and varicella-zoster virus DNA were negative. CSF cultures grew no bacteria or fungi. A human immunodeficiency virus test was negative. The magnetic resonance imaging results of the brain were normal. Figure 1 shows the clinical course and treatment of the patient. He was suspected of having meningitis and was empirically treated with micafungin (150 mg/d) and cefozopran (4 g/d), followed by meropenem (3 g/d), acyclovir (2250 mg/d), and amphotericin B (350 mg/d). However, cryptococcal antigen was detected in CSF (titers, 1:16) and serum on day 6 (Table 1). We discontinued the treatment with ruxolitinib and continued the administration of amphotericin B (350 mg/d), and the patient's condition improved until day 10. Amphotericin B was used until day 37, followed by administration of fluconazole (400 mg/d). The patient was on continuous therapy at the time of this report.

**Table 1:**
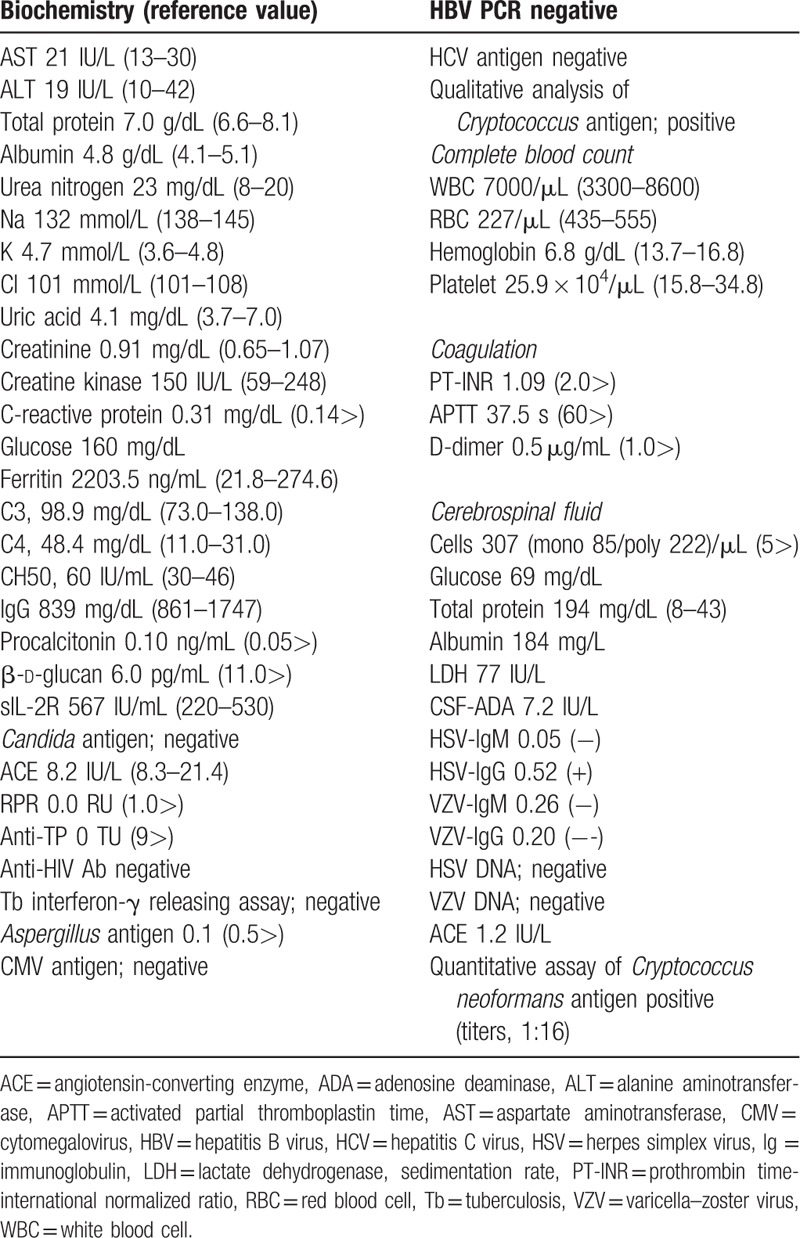
Laboratory data.

## 3 Discussion

Ruxolitinib is a selective JAK 1/2 inhibitor that has been approved for the treatment of MF and PV. The JAK/STAT pathway plays an important role in hematopoiesis and the immune response in vivo.^[1]^ After engagement of the receptor by the corresponding ligand, JAK becomes activated via phosphorylation, followed by JAK/STAT pathway activation.^[8]^ Activated STATs dimerize and translocate to the nucleus, where they regulate transcription and release proinflammatory cytokines and growth factors, including erythropoietin, granulocyte macrophage colony-stimulating factor, and thrombopoietin.^[9]^ In patients with MF, gene mutations, such as those in JAK2 and MPL, are in a constant phosphorylated state, independent of the binding of ligand to its receptor.^[10,11]^ Excess release of proinflammatory cytokines and growth factors triggers the systemic symptoms of MF and ineffective hematopoiesis.^[12]^ Blockage of JAK1 mainly improves systemic symptoms via a reduction in proinflammatory cytokines, and blockage of JAK2 mainly improves splenomegaly and anemia via a reduction in growth factors and prevents ineffective hematopoiesis.^[9,13–15]^ However, some opportunistic infections related to ruxolitinib have been reported previously.^[16–25]^

The present case involved cryptococcal meningitis in a patient treated with ruxolitinib. To the best of our knowledge, only 2 cases of cryptococcal meningitis in ruxolitinib-treated patients have been previously reported.^[16,17]^Table 2 summarizes cryptococcal meningitis associated with ruxolitinib, including our case, and 2 clinical features were found. First, the opening pressure of lumbar puncture in the present case was not high, although raised CSF pressure is one of the typical clinical features of cryptococcal meningitis. Half of patients with cryptococcal meningitis show a CSF opening pressure over 250 mm H_2_O; additionally, a quarter of patients show an extremely high pressure over 350 mm H_2_O.^[7]^ The mechanism of high CSF pressure is presumed to block CSF reabsorption by live or dead organisms, with shed cryptococcal polysaccharide at the level of the arachnoid granulations and other CSF reabsorption sites. Loyse et al^[26]^ reported that arachnoid granulation tissue contains many fungal cells in comparison with other sites of the brain, and high numbers of organisms are associated with increased antemortem CSF pressure. Bicanic et al^[27]^ reported that high CSF pressure in cryptococcal meningitis is associated with the phenotype of an infectious *Cryptococcus neoformans* strain and host factors other than the numbers of fungal cells. Among 3 patients with cryptococcal meningitis associated with ruxolitinib, 2 had normal opening CSF pressure (opening CSF pressure was not describe in 1 patient) (Table 2). Among the 3 cases, no trend was observed in CSF findings, such as the degree of pleocytosis and protein elevation. To the best of our knowledge, there has been no report of cryptococcal meningitis associated with ruxolitinib showing the numbers of fungal bodies in a postmortem study. Because only 2 cases have previously been reported, more studies are needed to confirm whether a normal CSF pressure is one of the features of cryptococcal meningitis associated with ruxolitinib or just the finding in our case.

**Table 2:**
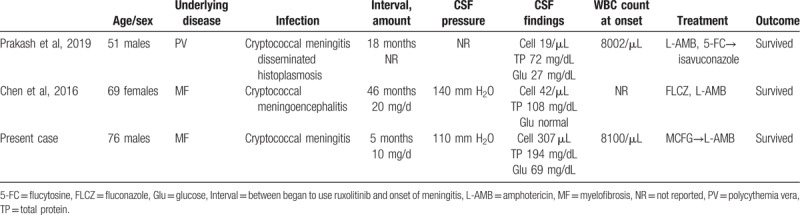
Cryptococcal meningitis associated with ruxolitinib.

Second, the outcome of the present case was relatively good compared to typical cryptococcal meningitis.^[7]^ The 2 previous cases of cryptococcal meningitis associated with ruxolitinib also showed good clinical outcomes (Table 2).^[16,17]^ In the present case, ruxolitinib was discontinued on day 6, and in 1 previous report, ruxolitinib was discontinued when fungal infection was found (in the other case, whether ruxolitinib was discontinued was not described). Hirano et al^[18]^ suggested that ruxolitinib administration should be discontinued if possible; otherwise, the treatment with ruxolitinib may be ineffective against a pulmonary cryptococcus infection. Additionally, in our case, discontinuing ruxolitinib may lead to a good outcome; therefore, as ruxolitinib may impact the immune response against cryptococcosis, ruxolitinib should be discontinued immediately when cryptococcal meningitis is suspected.

A phase III study of ruxolitinib reported that reactivation of tuberculosis and herpes zoster virus were the predominant opportunistic infections observed with ruxolitinib.^[28]^ Since that time, some cases of opportunistic infection associated with ruxolitinib have been reported.^[16–25]^ Dioverti et al^[29]^ published a review of 32 cases identified as opportunistic infections associated with ruxolitinib. Although the majority of cases reported were reactivations of tuberculosis (34%), several fungal infections were also reported (22%), and cryptococcus was the most frequently reported fungus. In a phase II, phase III, and long-term extension clinical trial with 5671 patients treated with tofacitinib, another JAK inhibitor approved for the treatment of adult patients with rheumatoid arthritis, cryptococcal infections were also reported (2 pulmonary infections and 1 case of meningitis).^[30]^

Many studies have tried to elucidate the mechanism by which ruxolitinib impacts the immune system. A review of the literature conducted by Manduzio indicated that the immunological derangement of ruxolitinib is mainly based on T cells, DCs, and NK cell defects.^[31]^ Heine et al^[4]^ reported that ruxolitinib affects the function and phenotype of DCs, leading to impaired T-cell activation. Ostoji et al reported that ruxolitinib suppresses cell-mediated immunity by inhibiting the T-helper lymphocyte 1 response and reducing the production of interferon-γ.^[32]^ The host defense against *C neoformans* infection is associated with cell-mediated immunity, especially accomplished by the combined action of activated macrophages, NK cells, and T cells.^[33]^ In addition, Hardison et al^[34]^ reported that STAT1 and signaling through the JAK/STAT pathway play an important role in the protective response against cryptococcosis via STAT1-mediated classical macrophage activation. In the present case, suppression of anticryptococcal responses was likely to induce the development of cryptococcal meningitis, as in the previously reported cases of cryptococcal infection.^[16–19,22]^ Ruxolitinib-associated opportunistic infections are not time-dependent and may occur any time after initiation of the drug.^[28]^ However, whether this effect is dose dependent is still controversial.^[16]^

In conclusion, we report a case of cryptococcal meningitis associated with ruxolitinib. Ruxolitinib administration is known to lead to opportunistic infections,^[28]^ and thus, it can cause cryptococcal meningitis, as the incidence of cryptococcal meningitis increases in patients with immunosuppressant conditions.^[7]^ Physicians should consider the possibility of cryptococcal meningitis in patients receiving ruxolitinib and discontinue the drug if possible when high-grade fever persists even in the absence of headache. Because ruxolitinib is a relatively new drug, further accumulation of clinical experience to monitor possible side effects is needed.

## Acknowledgment

The authors thank Dr Honoka Arai and Dr Yuko Nakamura, Department of Hematology and Oncology, Dokkyo Medical University Hospital, for their assistance with this study.

## Author contributions

**Conceptualization:** Daisuke Tsukui, Hiroaki Fujita.

**Writing – original draft:** Hiroaki Fujita, Keisuke Suzuki.

**Writing – review and editing:** Hiroaki Fujita, Koichi Hirata.

Hiroaki Fujita orcid: 0000-0003-2184-7916.
